# Supplementary material for: Novel Genetic Lineages of Rickettsia helvetica Associated with Ixodes apronophorus and Ixodes trianguliceps Ticks
Source: Microorganisms. 2023 May 5;11(5):1215. doi: 10.3390/microorganisms11051215 (PMC10223337; doi:10.3390/microorganisms11051215)
Supplement: Supplementary file 1 [file microorganisms-11-01215-s001.zip › Rar-Table S2.docx]

Table S2. Detection of *Rickettsia* spp. in ticks collected from individual rodents (without molting)

| Rodent | Rodent species | Tick species | No of ticks (L/N/Ad) | No of ticks (L/N/Ad) containing DNA of tested agents* | | | | |
| --- | --- | --- | --- | --- | --- | --- | --- | --- |
|  |  |  |  | All Rickettsia  species | R.tar | R.helv | R.ural | Mixed infection |
| BU 70 | M.glar. | I.pers | 25 / 1 / 0 | 25 / 1 / 0 | 25 / 1 / 0 | - | - | - |
|  |  | I.apr | 2 / 1 / 0 | 2 / 0 / 0 | 1 / 0 / 0 | 1^3^ / 0 / 0 | - | - |
| BU 75 | M.glar. | I.pers | 1 / 0 / 0 | 1 / 0 / 0 | 1 / 0 / 0 | - | - | - |
|  |  | I.apr | 1 / 1 / 0 | 0 / 1 / 0 | - | 0 / 1^3^ / 0 | - | - |
|  |  | I.tr | 4 / 1 / 0 | 4 / 1 / 0 | - | 4^2^ / 0 / 0 | 0 / 1 / 0 | - |
| BU 81 | M.glar. | I.apr | 3 / 0 / 0 | - | - | - | - | - |
| BU 93 | M.glar. | I.apr | 0 / 1 / 0 | 0 / 1 / 0 | - | 0 / 1^3^ / 0 | - | - |
| BU 94 | M.glar. | I.apr | 1 / 0 / 0 | - | - | - | - | - |
|  |  | I.tr | 5 / 0 / 1 | 5 / 0 / 1 | - | 5^2^ / 0 / 0 | 0 / 0 / 1 | - |
| BU 95 | M.glar. | I.pers | 1 / 0 / 0 | 1 / 0 / 0 | 1 / 0 / 0 | - | - | - |
|  |  | I.apr | 2 / 0 / 1 | 1 / 0 / 0 | - | 1^3^ / 0 / 0 | - | - |
| BU 101 | M.glar. | I.pers | 2 / 2 / 0 | 0 / 2 / 0 | 0 / 2 / 0 | - | - | - |
|  |  | I.tr | 1 / 0 / 0 | - | - | - | - | - |
| BU 123 | M.glar. | I.apr | 1 / 0 / 0 | 1 / 0 / 0 | - | 1^3^ / 0 / 0 | - | - |
|  |  | I.tr | 1 / 0 / 0 | - | - | - | - | - |
| BU 125 | M.glar. | I.pers | 2 / 0 / 0 | 2 / 0 / 0 | 1 / 0 / 0 |  |  | 1* / 0 / 0  *Rt+Rrao |
| BU 127 | M.glar. | I.pers | 2 / 0 / 0 | 2 / 0 / 0 | 2 / 0 / 0 | - | - | - |
|  |  | I.tr | 3 / 2 / 1 | 1 / 2 / 1 | - | - | 1 / 2 / 1 | - |
| BU 84 | M.rutil. | I.pers | 3 / 0 / 0 | - | - | - | - | - |
|  |  | I.apr | 2 / 0 / 0 | 1 / 0 / 0 | - | 1^3^ / 0 / 0 | - | - |
| BU 86 | M.rutil. | I.pers | 4 / 0 / 0 | 2 / 0 / 0 | 2 / 0 / 0 | - | - | - |
| BU 100 | M.rutil. | I.apr | 3 / 0 / 0 | 1 / 0 / 0 | - | 1^3^/ 0 / 0 | - | - |
| BU 132 | M.rutil. | I.pers | 7 / 0 / 0 | 6 / 0 / 0 | 6 / 0 / 0 | - | - | - |
| BU 79 | Mi.oec | I.pers | 2 / 0 / 0 | 2 / 0 / 0 | 2 / 0 / 0 | - | - | - |
|  |  | I.apr | 10 / 0 / 3 | 10 / 0 / 3 | - | 10^3^ / 0 / 2^3^ | - | 0 / 0 / 1**  **Rt+Rhelv^3^ |
| BU 83 | Ap.agr. | I.pers | 1 / 0 / 0 | - | - | - | - | - |
|  |  | I.apr | 2 / 0 / 0 | 1 / 0 / 0 | - | 1^3^ / 0 / 0 | - | - |
| BU 89 | Ap.agr. | I.apr | 1 / 1 / 0 | 1 / 1 / 0 | - | 1^3^ / 1^3^ / 0 | - | - |
| BU 110 | Ap.agr. | I.pers | 3 / 0 / 0 | 3 / 0 / 0 | 3 / 0 / 0 | - | - | - |
|  |  | I.apr | 1 / 0 / 0 | - | - | - | - | - |
|  |  | I.tr | 2 / 0 / 0 | - | - | - | - | - |
| BU 117 | Ap.agr. | I.apr | 0 / 0 / 1 | 0 / 0 / 1 | - | 0 / 0 / 1^3^ | - | - |
| BU 129 | Ap.agr. | I.apr | 0 / 0 / 1 | 0 / 0 / 1 | - | 0 / 0 / 1^3^ | - | - |
| BU 155 | Ap.agr. | I.pers | 1 / 0 / 0 | 1 / 0 / 0 | 1 / 0 / 0 | - | - | - |
|  |  | I.tr | 0 / 0 / 1 | - | - | - | - | - |
| BU 158 | Ap.agr. | I.pers | 1 / 0 / 0 | 1 / 0 / 0 | 1 / 0 / 0 | - | - | - |
|  |  | I.apr | 1 / 0 / 0 | - | - | - | - | - |
|  |  | I.tr | 0 / 1 / 0 | 0 / 1 / 0 | - | - | 0 / 1 / 0 | - |
| BU 103 | Ar.amph | I.apr | 0 / 0 / 3 | 0 / 0 / 2 |  | 0 / 0 / 2^3^ |  |  |
| BU 104 | Ar.am. | I.apr | 1 / 0 / 1 | 1 / 0 / 1 |  | 1^3^ / 0 / 1^3^ |  |  |
| BU 114 | Ar.am. | I.apr | 2 / 1 / 0 | 2 / 1 / 0 | - | 2^3^ / 1 / 0 | - | - |
| BU 116 | Ar.am. | I.apr | 2 / 0 / 0 | 2 / 0 / 0 | - | 2^3^/ 0 / 0 | - | - |
| BU 136 | Ar.am. | I.pers | 0 / 1 / 0 | - | - | - | - | - |
| BU 145 | Ar.am. | I.apr | 11 / 0 / 0 | 10 / 0 / 0 | - | 10^3^ / 0 / 0 | - | - |
|  |  | I.tr | 1 / 0 / 0 | - | - | - | - | - |
| BU 146 | Ar.am. | I.apr | 1 / 0 / 0 | 1 / 0 / 0 | - | 1^3^ / 0 / 0 | - | - |
| **Total** |  | **I.pers** | **55 / 4 / 0** | **46 / 3 / 0** | **45 / 3 / 0** | **-** | **-** | **1* / 0 / 0**  ***Rt+Rrao** |
|  |  | **I.apr** | **47 / 5 / 10** | **34 / 4 / 8** | **1 / 0 / 0** | **33^3^ / 4^3^/ 7^3^** | **-** | **0 / 0 / 1****  ****Rt+Rhelv**^3^ |
|  |  | **I.tr** | **17 / 4 / 3** | **10 / 4 / 2** | **-** | **9^2^/ 0 / 0** | **1 / 4 / 2** | **-** |

* Including cases of mixed infection. Superscript numbers indicate the genetic lineage of *R. helvetica* in ticks.

Abbreviations: M.glar - *M. glariolus*; M.rutil - *M. rutilus*; Mi.oec - *Mi. oeconomus*; Ap.agr - *Ap.agrarius*; Ar.amph - Ar. *amphibious*; I.pers - *I. persulcatus*; I.apr – *I. apronophorus*; I.tr - *I. trianguliceps*; L – larvae; N – nymphs; Ad – adults; Rtar –“*Ca.* R. tarasevichiae”; Rhelv – *R. helvetica*; Rur - “*Ca.* R. uralica” ; Rrao – *R. raoultii*
